# Effects of reduced platelet count on the prognosis for patients with non-small cell lung cancer treated with EGFR-TKI: a retrospective study

**DOI:** 10.1186/s12885-020-07650-2

**Published:** 2020-11-26

**Authors:** Lu Xu, Fangzhou Xu, Haobo Kong, Meiling Zhao, Yuanzi Ye, Yanbei Zhang

**Affiliations:** 1 Department of Geriatric Respiratory and Critical Care, Anhui Geriatric Institute, the First Affiliated Hospital of Anhui Medical University, Hefei, Anhui 230022 People’s Republic of China; 2 Department of Pathology, the First Affiliated Hospital of Anhui Medical University, Hefei, Anhui 230022 People’s Republic of China

**Keywords:** Platelet, EGFR-TKI, Non-small cell lung cancer, Progression-free survival, Overall survival

## Abstract

**Background:**

Progressive lung cancer is associated with abnormal coagulation. Platelets play a vital part in evading immune surveillance and angiogenesis in the case of tumor metastasis. The study aimed to analyze the predictive and prognostic effects of platelet count on non-small cell lung cancer (NSCLC) patients treated with epidermal growth factor receptor tyrosine kinase inhibitors (EGFR-TKIs).

**Methods:**

This study retrospectively analyzed the prognostic effects of platelets on 52 NSCLC patients with epidermal growth factor receptor (EGFR) mutant following EGFR-TKI treatment. Related data, together with the progression-free survival (PFS) and overall survival (OS) were collected before and after 2 cycles of treatments (60 days).

**Results:**

The anti-EGFR treatment markedly reduced the platelet count in 33 (63.5%) patients after 2 cycles of treatment. Multivariate Cox analysis revealed that, the decreased platelet count was closely correlated with the longer OS (HR = 0.293; 95%CI: 0.107-0.799; *p* = 0.017). Besides, the median OS was 326 days in the decreased platelet count group and 241 days in the increased platelet count group (HR = 0.311; 95%CI: 0.118-0.818; *P* = 0.018), as obtained from the independent baseline platelet levels and other clinical features.

**Conclusions:**

The platelet count may predict the prognosis for EGFR-TKI treatment without additional costs. Besides, changes in platelet count may serve as a meaningful parameter to establish the prognostic model for NSCLC patients receiving anti-EGFR targeted therapy.

## Background

Advanced cancer is frequently accompanied with overt or subclinical thrombosis, which is manifested as thromboembolism (TE), especially for venous thromboembolism (VTE), including deep vein thrombosis (DVT) and pulmonary embolism (PE) [1]. Under the control of mutated epidermal growth factor receptor (EGFR), renin-angiotensin system (RAS), methionine (MET) or other tumorigenic pathways, vascular endothelial growth factor (VEGF) and other mediators are over-expressed in lung cancer patients. This thereby induces the growth of exuberant blood vessels, the remarkable remodeling of vascular walls, the activation of coagulation system inside or outside the vascular lumen, and high incidence of intracavity occlusive thrombus [2].

Activated coagulation may also take place in the form of bleeding or mixed disorder like disseminated intravascular coagulation (DIC), or the more subtle subclinical laboratory changes (such as the elevated fibrin d-dimer levels, the prolonged prothrombin time, and the markedly reduced platelet count) [3]. TE is usually observed in adenocarcinoma patients at the time of diagnosing the cancer, whereas DIC is often detected at the advanced cancer stage that is associated with a short survival time. The frequency of TE may be underestimated, and the radiological characteristics of TE rarely present when diagnosing cancer patients with DIC or those at the terminal stage [4].

As the small discoid anuclear cell fragments, platelets exert key parts in hemostasis, which represents a frontline physiological response to acute tissue injury. Vascular disruption triggers the localized deposition of platelets, as well as the release and activation of platelet granular ingredients. In addition, the platelet-released molecules (PRMs) can further recruit and activate platelets, resulting in multi-cellular aggregates and restricting blood loss. The activated coagulation cascades jointly lead to thrombin generation, which thereby consolidates the increased thrombus through further promoting platelet activation and catalyzing fibrin formation [5].

Accumulated evidence demonstrates that, the development of lung cancer is closely correlated with the platelet-induced thrombus, which is considered as a candidate cancer biomarker. Platelets can be activated through the direct interactions between cancer cells, or via the indirect mediators released by cancer cells. In turn, the activated platelets invoke the phenotypic transformation of cancer cells by means of metastasis and angiogenesis [6]. On the other hand, thrombus formation is also involved in some oncogenic mutated genes, including the the protein-coding genes (such as EGFR, RAS) and non-coding RNA (microRNA). A series of gene mutations regulate multiple effectors in the coagulation system, like tissue factors, protease activated receptors (PAR-1 and PAR-2), coagulation factors (FII and FVII), as well as platelet function and fibrinolysis mediators [7, 8].

The platelet count is frequently regarded as one part of the routine laboratory tests, which can be utilized to predict thrombosis without inducing any additional costs. This study aimed to assay the predictive and prognostic effects of changes in platelet count on non-small cell lung cancer (NSCLC) patients receiving anti-EGFR targeted therapy, and to determine the anti-cancer efficacy of EGFR tyrosine kinase inhibitors (TKIs).

## Methods

### Patient enrollment

From March 2016 to March 2019, a total of 52 patients with advanced or recurrent NSCLC and EGFR mutations treated with EGFR-TKI at the First Affiliated Hospital of Anhui Medical University were retrospectively analyzed in this study. The criteria for the use of anti-EGFR therapy were as follows: (1) deletion in exon 19 (*n* = 23); (2) substitution in exon 21 (*n* = 14); (3) amplification of EGFR gene (*n* = 3); and (4) the remaining patients were qualified for treatment in the past era when there was no obligation to indicate the EGFR gene mutation status. In addition, patients with active infection, systemic corticosteroids, or haematological malignancy were excluded, since these conditions might affect the test values of hematological laboratory markers.

### Data collection

The following variables were extracted, including platelet count, D-dimer concentration, age, gender, clinical stage, histological diagnosis, smoking, EGFR mutation, other therapies (like thoracic surgery, chemotherapy, radiofrequency ablation, and radiotherapy), concomitant diseases (like cerebral infarction, arterial hypertension, diabetes mellitus, heart disease, chronic obstructive pulmonary disease), and the choice of TKIs (such as gefitinib, erlotinib, ectinib, or ositinib). Besides, routine assessments of complete blood count and coagulogram (including the platelet concentration, D-dimer concentration) were carried out before and after 2 cycles of therapy (first effective evaluation after 60 days of treatment). All clinical data were utilized for evaluating the therapeutic efficacy in combination with the chest Computed Tomography (CT) scan results in accordance with the Response Evaluation Criteria in Solid Tumors (RECIST, version 1.1). The primary endpoint was PFS calculated from the date of TKI initiation to the documented disease progression or death of any cause. OS was defined as the duration between the initiation of EGFR-TKI treatment and the date of death due to any cause.

### Statistical analysis

SPSS 22.0 statistical software package (SPSS Inc., Chicago, Illinois, USA) was adopted for data analysis. The abnormally distributed continuous parameters were expressed as median and quartiles. The platelet count after 2 cycles of treatment was compared to that with no treatment through the Wilcoxon’s test. Chi-square test was used to compare the correlation between different variables and patient prognosis. Univariate Cox regression analysis was applied to identify prognostic variables. Then, significant variables were incorporated into the multivariate Cox regression analysis to analyze independent influencing factors. Meanwhile, the survival curves were plotted according to the Kaplan-Meier method and log-rank test. A two-sided *P*-value of < 0.05 was deemed as statistically significant.

## Results

### Patients’ characteristics

A total of 52 advanced or recurrent NSCLC patients with common EGFR mutation were treated by EGFR-TKIs during the study period. Table 1 presents the detailed patient characteristics. There were 26 (50%) males and 26 (50%) females in the total cohort, with the median age of 62.5 years (Quartiles, 50.3-69). Besides, 82.9% patients were treated with chemotherapy, 6.4% with radiofrequency ablation, 4.3% with radiotherapy, 4.3%with thoracic surgery, and 2.1% with radioactive seed implantation. Of these patients, 14 had arterial hypertension, 9 had diabetes mellitus, 3 had heart disease, 3 had chronic obstructive pulmonary disease, and 3 had cerebral infarction. Additionally, 17 of these 52 cases had a history of smoking. The two-cycle TKI treatment resulted in the markedly reduced platelet count (*n* = 33, 63.5%), D-dimer content (*n* = 37, 71.2%). Table 1.

**Table 1:** Patients’ characteristics

Variables	N	%
**Age**		
Median	62.5	
Quartiles	50.3-69	
**Gender**		
Female VS Male	26:26	50: 50
**Clinical stage**		
III B VS IV	3:49	5.8: 94.2
**Histological diagnosis**		
Adenocarcinoma VS Others	51:1	98.1: 1.9
**Anti-EGFR therapy**		
Gefitinib	34	65.4
Erlotinib	7	13.5
Ectinib	9	17.3
Ositinib	2	3.8
**Other treatments**		
Thoracic surgery	2	4.3
Radiotherapy	2	4.3
Chemotherapy	39	82.9
Radiofrequency ablation	3	6.4
Radioactive seed implantation	1	2.1
**Concomitant diseases**		
Arterial hypertension	14	43.7
Diabetes mellitus	9	28.1
Heart disease	3	9.4
Chronic obstructive pulmonary disease	3	9.4
Cerebral infarction	3	9.4
**Smoking**		
Past/current	17	32.7
Never	35	67.3
**D-dimer content**		
Decrease VS Increase	37:15	71.2: 28.8
**Platelet count**		
Decrease VS Increase	33: 19	63.5: 36.5

### Evaluation of blood indicators in patients before and after treatment

Table 2 displays the detailed characteristics of platelet count and D-dimer content. After the two-cycle TKI treatment, the platelet count (*p* = 0.026) and D-dimer content (*p* = 0.007) were markedly reduced compared with those in non-treatment.

**Table 2:** The detailed characteristics of platelet count and D-dimer content during the first two-cycle treatment

Values	N	Before Treatment	After Treatment	Statistic(Z)	***P***-value
**Platelet count**	52				
Median		230	211	−2.222	0.026
Quartiles		172-281.8	159.75-236.5		
Decrease group	33				
Median		248	211	−5.012	0.000
Quartiles		192-335	159-233.5		
Increase group	19				
Median		172	211	−3.726	0.000
Quartiles		133-225	159-276		
**D-dimer content**	52				
Median		0.94	0.725	−2.687	0.007
Quartiles		0.53-2.72	0.31-1.415		
Decrease group	37				
Median		1.48	0.42	−5.303	0.000
Quartiles		0.53-3.24	0.245-1.145		
Increase group	15				
Median		0.88	1.22	−3.408	0.001
1Quartiles		0.47-0.95	1.00-2.09		

In our laboratory, 125-350(*10^9^/L) is adopted as the cut-off value for normal platelet; < 0.5 μg/ml is used as the cut-off value for normal D-dimer

Afterwards, patients were classified as the increase and decrease groups according to the blood indicator values. In the platelet decrease group, the median platelet count before treatment (248; 192-335) was higher than that after treatment (211; 159-233.5) (*P* <  0.001). For patients in the platelet increase group, the median platelet count was 172(133-225) before treatment and 211 (159-276) after anti-EGFR treatment, which was markedly increased (*P* <  0.001). Among patients in the D-dimer decrease group, the median D-dimer level after TKI treatment (0.42; 0.245-1.145) was evidently decreased compared with that before treatment (1.48;0.53-3.24) (*P* < 0.001). Similarly, patients in the D-dimer increase group had remarkably increased D-dimer levels (*P* = 0.001). Table 2.

### Comparison of prognostic factors within groups

Patients were divided into the PFS (+) group and the PFS (−) group according to whether they had progressed. Patients were divided into OS (+) and OS (−) groups according to whether they died or not.

Chi-square analysis results (Table 3) show that there is no difference between PFS (−) and PFS (+) for each indicator (*P* > 0.05). Moreover, results of Chi-square analysis (Table 4) indicated that, changes in platelet count before and after treatment were different in OS (−) and OS (+) (*P* < 0.05). Similarly, gender, smoking and the presence of other diseases were also statistically significant in both groups (*P* < 0.05). Tables 3 and 4.

**Table 3:** The difference of prognostic factors between PFS (−) group and PFS(+) group

Values	PFS (−)	PFS (+)	Statistic(χ^**2**^)	***P***-value
**N**	5	47		
**Platelet**			0.653	0.390
Increase	1(20%)	18(38.3%)		
Decrease	4(80%)	29(61.7%)		
**D-dimer**			0.211	0.549
Increase	1(20%)	14(29.8%)		
Decrease	4(80%)	33(70.2%)		
**Gender**			0.221	0.500
Male	2(40%)	24(51.1%)		
Female	3(60%)	23(48.9%)		
**Age**			0.221	0.500
≥ 62.5	2(40%)	24(51.1%)		
**<** 62.5	3(60%)	23(48.9%)		
**Smoking**			0.405	0.467
Past/current	4(80%)	31(66.0%)		
Never	1(20%)	16(34.0%)		
**Exon21**			2.038	0.153
No	5(100%)	33(70.2%)		
Yes	0(0%)	14(29.8%)		
**Exon19**			4.387	0.059
No	5(100%)	24(51.1%)		
Yes	0(0%)	23(48.9%)		
**Other diseases**			3.457	0.077
No	5(100%)	27(57.4%)		
Yes	0(0%)	20(42.6%)		
**Other treatment**				
No	1(20%)	19(40.4%)	0.797	0.354
Yes	4(80%)	28(59.6%)		

*PFS* progression-free survival, *PFS (+)* progression group, *PFS (−)* No-progression group

**Table 4:** The difference of prognostic factors between OS (−) group and OS (+) group

Values	OS (−)	OS (+)	Statistic(χ^**2**^)	***P***-value
**N**	34	18		
**Platelet**			4.294	0.039
Increase	9(26.5%)	10(55.6%)		
Decrease	25(73.5%)	8(44.4%)		
**D-dimer**			1.353	0.337
Increase	8(23.5%)	7(38.9%)		
Decrease	26(76.5%)	11(61.1%)		
**Gender**			8.497	0.008
Male	12(35.3%)	14(77.8%)		
Female	22(64.7%)	4(22.2%)		
**Age**			3.059	0.072
≥ 62.5	14(41.2%)	12(66.7%)		
< 62.5	20(58.8%)	6(33.3%)		
**Smoking**			6.540	0.013
Past/current	27(79.4%)	8(44.4%)		
Never	7(20.6%)	10(55.6%)		
**Exon21**			0.575	0.519
No	26(76.5%)	12(66.7%)		
Yes	8(23.5%)	6(33.3%)		
**Exon19**			0.001	0.605
No	19(55.9%)	10(55.6%)		
Yes	15(44.1%)	8(44.4%)		
**Other diseases**			5.967	0.016
No	25(73.5%)	7(38.9%)		
Yes	9(26.5%)	11(61.1%)		
**Other treatment**			1.549	0.172
No	11(32.4%)	9(50%)		
Yes	23(67.6%)	9(50%)		

*OS* overall survival, *OS (−)* survival group, *OS (+)* death group

### Prognostic factors in patients treated with EGFR-TKI

As shown in Table 5, no variables were associated with PFS in patients receiving EGFR-TKI, as shown in Table 3.

**Table 5:** Univariate and multivariate Cox analysis of PFS among patients who received EGFR-TKI treatment

	Univariate analysis			Multivariate analysis		
	HR	95%CI	*P*	HR	95%CI	*P*
Platelet						
(Decrease VS Increase)	0.592	0.325-1.080	0.087			
D-dimer						
(Decrease VS Increase)	0.733	0.387-1.391	0.342			
Male VS Female	0.728	0.399-1.326	0.299			
Age (≥62.5 vs. others)	1.104	0.617-1.976	0.738			
Substitution in exon 21						
(Yes vs. No)	0.934	0.489-1.787	0.837			
Deletion in exon 19						
(Yes vs. No)	1.392	0.774-2.502	0.269			
Smoking(Yes vs. No)	1.360	0.728-2.539	0.335			
Other treatment						
(Yes vs. No)	0.691	0.377-1.266	0.231			
Other diseases						
(Yes vs. No)	1.254	0.695-2.263	0.452			

*PFS* progression-free survival, *HR* hazard ratio

As shown in Table 6, Variables with *P* < 0.05 in univariate analysis were included in multivariate analysis. Multivariate Cox analysis revealed that patients with decreased platelet count (HR = 0.293; 95%CI: 0.107-0.799; *p* = 0.017), and male (HR = 0.195; 95%CI: 0.042-0.914; *p* = 0.038) had longer OS. And the presence or absence of smoking and other diseases had no significant effect on survival. Tables 5 and 6**.**

**Table 6:** Univariate and multivariate Cox analysis of OS among patients who received EGFR-TKI treatment

	Univariate analysis			Multivariate analysis		
	HR	95%CI	*P*	HR	95%CI	*P*
Platelet						
(Decrease VS Increase)	0.311	0.118-0.818	0.018	0.293	0.107-0.799	0.017
D-dimer						
(Decrease VS Increase)	0.538	0.204-1.416	0.209			
Male VS Female	0.207	0.059-0.724	0.014	0.195	0.042-0.914	0.038
Age (≥62.5 vs. others)	1.937	0.716-5.240	0.193			
Substitution in exon 21						
(Yes vs. No)	1.008	0.355-2.867	0.988			
Deletion in exon 19						
(Yes vs. No)	1.150	0.443-2.987	0.774			
Smoking(Yes vs. No)	2.931	1.112-7.730	0.030	0.695	0.199-2.422	0.568
Other treatment						
(Yes vs. No)	0.500	0.192-1.300	0.155			
Other diseases						
(Yes vs. No)	2.92	1.079-7.901	0.035	2.796	0.970-8.059	0.057

*OS* overall survival, *HR* hazard ratio

### Survival analysis

**Fig. 1:**
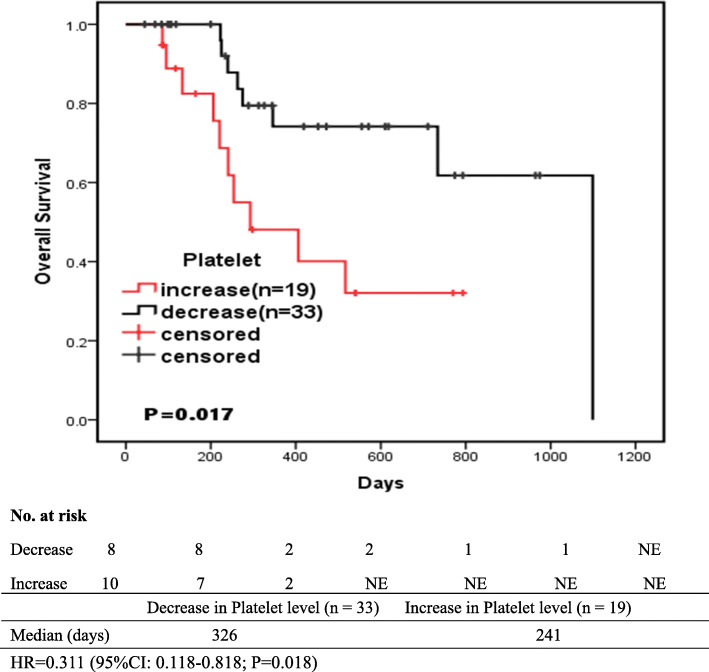
Kaplan-Meier-estimated OS on stratifying by the platelet count {decrease (*N* = 33) vs. increase(*N* = 19)}

A total of 52 patients were observed during the follow-up period, and the median OS among decrease group of platelet was 326 (211.5-614) days. And, the OS values were observed in increase group, with the median OS of 241 (117-517) days. The OS was remarkably extended in patients with decreased platelet count (Fig. 1). The difference in overall survival between the two groups was statistically significant(*P* < 0.05). Figure 1 shows the proportions of patients with no progression at various time points between the platelet decrease and increase groups.

**Fig. 2:**
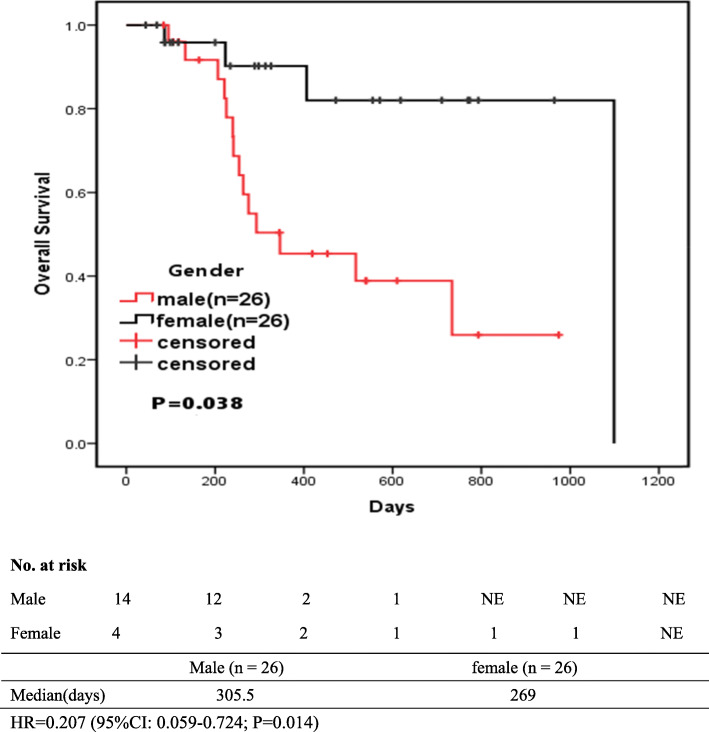
Kaplan-Meier-estimated OS on stratifying by gender {male (*N* = 26) vs. female (*N* = 26)}

In the meantime, the OS values were observed in male patients, with the median OS of 305.5 (103.5-641.25) days. Male Patients had a longer OS (Fig. 2). Figure 2 shows the survival rates of male patients and female patients at different time points.

## Discussion

In recent years, the more advanced anticancer therapy has been applied in clinical practice thanks to the development of modern oncology, including the advances in genetics, genomics and proteomics. This results in the development of three types of oncologic biomarkers, namely, the predictive, prognostic and early response biomarkers [9]. To search for the appropriate anticancer treatment, some NSCLC patients harboring various genetic mutations have been enrolled for investigation and assay [10]. Recent studies indicate that, paraneoplastic thrombocytosis may serve as a factor to predict the dismal prognosis for lung cancer [11, 12]. As suggested by our results, the platelet count was the most potent differentiating factor for OS (Tables 4 and 6), and EGFR-TKI treatment induced the remarkably reduced platelet count among NSCLC patients (Tables 1 and 2). Consistent with previous trial data on osimertinib which showed high efficacy over 1st and 2nd generation EGFR-TKI [13] yet is associated with decrease in platelet count [14].

Patients with advanced lung cancer may confront more risks related to the thromboembolic complications [1]. In addition, it is suggested in the international clinical practice guidelines that, primary antithrombotic prophylaxis, should be taken into consideration for patients with locally advanced or metastatic lung cancer that receive chemotherapy [15]. According to recent studies, the oncogenic events in cancer cells (such as the expression of mutant K-ras, EGFR) lead to the increased TF levels and activity, which thereby promoting tumor aggressiveness, angiogenesis, and hypercoagulability. Thus, the overt or cryptic abnormalities of coagulation parameters are detected in about 50% cancer patients as well as 90% with metastatic diseases [16–18].

TF is suggested in recent study to trans-activate EGFR and regulate gene expression [19, 20]. Typically, the TF-induced EGFR activation is closely correlated with the procoagulant activity of cancer cells, which is achieved through the Nuclear transcription factor activates protein-1 (AP-1) transcriptional activation [21]. Under such situations, TF binds to FVIIa, an enzyme active form of FVII, and triggers the production of other coagulation-related proteases, such as FXa and FIXa. Of them, FXa cooperates with FVa to cleave the circulating prothrombin for evoking the thrombin (FIIa) activity, which is required for activating platelets. Besides, it can set off the amplification phase of the hemostatic process through generating several active clotting factors, including FVIIIa and FXIa [22]. Even in the asymptomatic cases, coagulopathy can be inferred from laboratory findings, which is possibly accompanied with chronically activated coagulation, like the increased levels of circulating FVIIa, prothrombin fragment 1 + 2, and fibrinopeptides A and B, together with the altered (increased or decreased) platelet count [23].

Tumour metastasis is dependent on the interactions between tumour cells and the host circulatory microenvironments, such as the lymphatic vessels and targeted tissues; moreover, the platelet levels also support tumor metastasis [24]. At the molecular biology level, the malignant grade of tumor is associated with the levels of platelets and cytokines, for example, the VEGF, platelet-derived growth factor (PDGF), transforming growth factor β (TGF-β), and interleukin 6 (IL-6) [25]. Lim et al. demonstrated in their study that, the ratio of platelet combined with lymphocyte to monocyte might be adopted to predict the short survival for stage IV NSCLC patients [26]. In general, platelets are known to be up-regulated among lung cancer patients, and the high platelet count may imply the dismal prognosis [12]. Therefore, the current retrospective study was carried out to determine whether the decreased platelet count among lung cancer patients predicted the efficacy of EGFR-TKI treatment. According to our results, the EGFR-TKI treatment exhibited positive prognostic effect, along with the decreased platelet count (Table 2 and Fig. 1). It seemed that, such treatment indicated a novel approach for future studies concerning the anti-cancer biomarkers, including the anti-EGFR treatment.

Interestingly, the circulating platelets containing hepatocyte growth factor (HGF) and TGF-β may induce resistance to EGFR-TKIs, which is achieved through activating the bypass signaling pathway or the epithelial to mesenchymal transition (EMT) [27–30]. Miyata K et al. demonstrated that PDGF activated EGFR [31]. EGF is the only growth factor that is reported as the EGFR ligand in platelets [24]. EGF can directly control tumor disease and anticancer therapy by restoring the balance between prothrombotic and fibrinolytic processes.

Moreover, some existing data show that, the longer PFS is not correlated with the longer OS among cancer patients who receive targeted drug therapy, like the anti-EGFR therapy [32]. On this account, our findings had definite clinical values, since the same results were found. During the treatment, OS were markedly prolonged with the decrease in platelet count. However, the change in PFS was not obvious (Tables 3 and 5). Perhaps the association between decreased platelet count and PFS can be more fully analyzed as the sample size increases.

It is suggested in some study that, a pathogenic feedback loop may be operative between platelets and tumor cells, along with the reciprocal interactions between tumor growth/metastasis and thrombocytosis/platelet activation. Specific molecular pathways have been identified, in which tumors can stimulate the production and activation of platelets, and this in turn will promote tumor growth and metastasis [12]. Based on certain clinical data, aspirin has been used to evaluate the effect of anti-platelet agents on cancer survival [6]. Specifically, a recent large, randomized cardiovascular prevention trial shows that, cancer patients with sufficient aspirin administration are associated with lower risks of cancer metastasis, which is achieved through inhibiting the platelet function [33]. Antiplatelet therapy combined with conventional targeted antitumor therapy should be considered in the future, even though its efficacy has not been unequivocally proven yet.

In addition, our study showed that the OS of male patients receiving EGFR-TKIS was relatively longer (Fig. 2). However, previous studies have shown that PFS and OS of females are longer than that of men [34], and more studies have found that gender has no correlation with PFS and OS [35, 36]. Therefore, it may be the insufficient sample size that leads to the deviation of results, which requires further research.

Some limitations should be noted in this study. Firstly, this was a retrospective study with a small sample size; Therefore, a prospective study with a greater sample size is required to validate our results. Secondly, the high censoring rate at the end of the follow-up had hampered the accurate estimation of OS. Thirdly, because the sample size was insufficient and the final outcome of lung cancer patients was progression, selection deviation was caused, which may have affected the results of the study on the correlation between platelet count and PFS.

## Conclusions

Our findings suggest that, the decreased platelet count seems to serve as a significant predictor for OS among NSCLC patients receiving EGFR-TKIs. Changes in platelet count may serve as a meaningful parameter to establish the prognostic model for patients receiving targeted therapy. Our results reveal a novel underlying mechanism of adjuvant therapy associated with the functional status of platelets.

## Data Availability

The datasets generated and/or analyzed during the current study are not publicly available due owing to data privacy policy at our facility, but are available from the corresponding author on reasonable request.

## Abbreviations

NSCLC: Non-small cell lung cancer
EGFR: Epidermal growth factor receptor
EGFR-TKIs: Epidermal Growth Factor Receptor tyrosine kinase inhibitors
PFS: Progression-free survival
OS: Overall survival
TE: Thromboembolism
VTE: Venous thromboembolism
HR: Hazard ratio
DVT: Deep vein thrombosis
PE: Pulmonary embolism,
VEGF: Vascular endothelial growth factor
microRNA: Non-coding RNA
DIC: Disseminated intravascular coagulation
MET: Methionine
PRMs: Platelet-released molecules
RAS: Renin-angiotensin system
PAR: Protease activated receptors
ALT: Alanine aminotransferase
AST: Aspartate aminotransferase
CT: Computed Tomography
RECIST: Response Evaluation Criteria in Solid Tumors
AP-1: Nuclear transcription factor activates protein-1
PDGF: Platelet-derived growth factor
IL-6: Interleukin 6
TGF-β: Transforming growth factor β
EMT: Mesenchymal transition
HGF: Hepatocyte growth factor
